# Challenge of gastro-intestinal stromal tumor management in low-income countries: example of Benin

**DOI:** 10.1186/s12957-022-02709-9

**Published:** 2022-12-01

**Authors:** Dansou Gaspard Gbessi, Freddy Houéhanou Rodrigue Gnangnon, Aboudou Raïmi Kpossou, Pacifique Prudent Gbetchedji, Falilatou Seidou, Yacoubou Imorou Souaïbou, Setondji Gilles Roger Attolou, Ismaïl Lawani, Marie-Christel Laleye, Flore Gangbo, Francis Moïse Dossou, Jean Sehonou, Delphin Kuassi Mehinto

**Affiliations:** 1 Department of Visceral Surgery, National University Hospital Hubert Koutoukou Maga (CNHU-HKM), Cotonou, Republic of Benin; 2 Department of Hepato-Gastroenterology, National University Hospital Hubert Koutoukou Maga (CNHU-HKM), Cotonou, Republic of Benin; 3 Department of Pathology, Faculty of Health Sciences/ Abomey-Calavi University (FSS/UAC), Cotonou, Republic of Benin; 4 Department of General Surgery, Departmental University Hospital Oueme-Plateau (CHDU-OP), Porto-Novo, Republic of Benin

**Keywords:** GIST, Access to treatment, Surgery, Imatinib, Low-income country, Benin republic

## Abstract

**Background:**

GISTs are rare tumors but the most frequent mesenchymal tumors of the digestive tract. Diagnosis and treatment are challenging in low-income countries due to relatively poor access to immunohistochemistry and targeted therapy. In Africa, there are few studies about it. Imatinib, an oral targeted therapy, has been available in Benin since 2010 and free since 2016. This study describes the diagnosis and therapeutic management of GIST in Cotonou, Benin.

**Methods:**

This is a descriptive cross-sectional study, with retrospective data collection over a 10-year period from 2010 to 2020, focused on patients with histological confirmed gastro-intestinal stromal tumor (GIST). Cases were identified using the registry database and the archival files of the Hubert Koutoukou Maga National University Hospital of Cotonou (CNHU-HKM).

**Results:**

Fifteen GISTs were identified during the study period. The median age was 52 and the sex ratio was 2:1 (10 males and 5 females). The most frequent symptom was abdominal pain (*n* = 12). Delay in care seeking after onset of symptoms ranged from 24 h to 15 years. The most common site for GISTs was the stomach (*n* = 8). The median tumor size was 11 cm and the majority (*n*=10) was metastatic or locally advanced at the time of diagnosis. The tumors were often spindle-shaped at histology (*n* = 13) and the majority expressed KIT (*n* = 14). Most of the tumors (*n* = 12) were at high risk of recurrence according to the Joensuu scoring system. The availability of imatinib has improved the outcome of GIST with response in all cases it was used in neoadjuvant setting (*n* = 7).

**Conclusion:**

GISTs are rare tumors and preferentially affect the stomach in Cotonou). Most of the tumors were large, unresectable at the time of diagnosis and at high risk of recurrence. Access to imatinib has revolutionized the management of those tumors in our country.

## Background

Gastrointestinal stromal tumors (GISTs) are rare tumors derived from Cajal cells or from one of their precursors. Their incidence is estimated 15 cases/million inhabitants/year [1–3]. However, they represent the most frequent mesenchymal tumors of the digestive tract [1]. GISTs account for 80 % of mesenchymal tumors, 5 % of sarcomas and less than 1 % of tumors of the digestive tract. Immunohistochemistry reveals often the expression of the CD117/KIT + (95%) and DOG-1 + (95%) markers. Clinically, GISTs remain silent for a long time until they reach a large size [2]. Although the potentially curative treatment for GISTs is surgical resection, their management has been revolutionized by the approval of imatinib as an adjuvant and neoadjuvant therapy [3–5]. Few studies have been made on GIST in Africa and to the best of our knowledge, no case series has been published in Benin [6, 7]. Tumors are often diagnosed at a later stage in Africa [8, 9]. Access to treatment was difficult in Africa especially in Benin because of the cost of imatinib [7, 10]. The availability of imatinib in Benin, thanks to the Glivec International Patient Assistance Program (GIPAP), over the past 5 years has revolutionized the management of this disease [11]. The Hubert Koutoukou Maga National University Hospital of Cotonou (CNHU-HKM) is one of the most important hospitals in Benin. It is the only one capable of ensuring the continuum of care for patients with GIST (availability of imatinib and digestive surgical oncology). Our aim is to describe the diagnosis, therapeutic management, and outcome of GIST since the availability of imatinib in our country.

## Methods

This is a descriptive cross-sectional study, with retrospective data collection at the Hubert Koutoukou Maga National University Hospital of Cotonou (CNHU-HKM), a major center for cancer treatment in Benin.

Cases were identified using the registry database and the archival files of the Hubert Koutoukou Maga National University Hospital of Cotonou (CNHU-HKM). All GIST cases managed from January 2010 to January 2020 were screened by reviewing their medical report. We included medical reports of all patients having the definitive diagnosis of GIST with a histological and immunohistochemical proof. We excluded patients with an incomplete medical report and those who presented a diagnostic discrepancy on histopathology and immunohistochemistry. Joensuu’s modified National Institute of Health (NIH) scoring system was used to assess risk of recurrence [3]. Vital status of cases (whether dead or alive at the closing date) was obtained by active methods. Cases whose vital status could not be confirmed at the end of this procedure were called when a phone number was available. When no further information could be obtained, home visits were made.

Data entry was made in an Epidata 4.6 software form. Data analysis was planned with Epidata Analysis software version 3.0.0.1. Given the small sample size, the statistics remains only descriptive.

## Results

### Clinical and diagnostic aspects

**Fig. 1:**
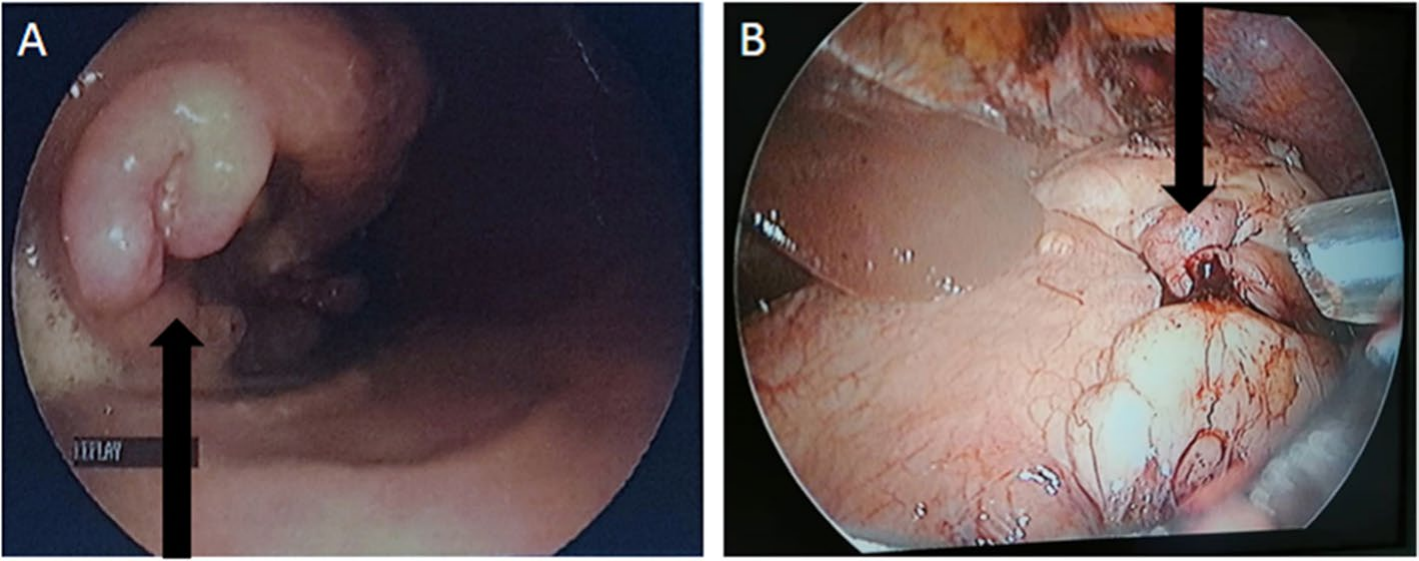
**A** Endoscopic view of a gastric GIST in a 66-year-old patient. **B** Laparoscopic view of a large gastric GIST (black arrow) invading the omental bursa in a 66-year-old patient

**Fig. 2:**
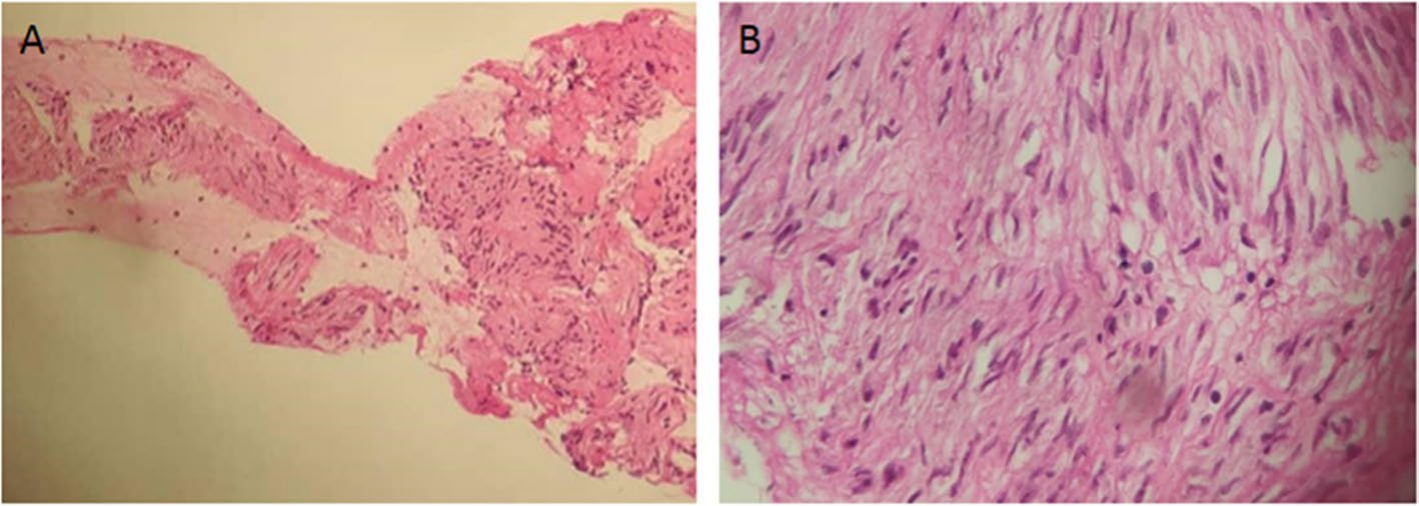
**A** Histological appearance of a gastric GIST in a 66-year-old patient zoom × 10, **B** zoom × 40

Fifteen GIST cases were identified during the study period. The median age was 52 (39 - 82 years). A male preponderance was noted with a sex ratio of 2:1 (10 males and 5 females). Delay in care seeking after onset of symptoms ranged from 24 h to 15 years. Most of the patients (*n* = 9) sought a medical consultation after one month. All patients were symptomatic, and the most frequent symptoms were abdominal pain (*n* = 12) followed by abdominal mass (*n* = 10) (Fig. 1). The stomach was the most common site for GISTs (*n*=8) followed by the large bowel (*n* = 3), then the small intestine (*n* = 2), and finally, the omentum and the mesentery with one case, each. Endoscopic examination had been performed in 7 patients and led to the detection of the tumor in 5 cases (Fig. 2). The endoscopic lesions were an ulceration (*n* = 2) and an ulcerative budding lesion (*n* = 3). Endoscopic biopsy was performed in three cases and led to the diagnosis of GIST in all cases. Diagnostic laparoscopy was performed in two cases. The remaining 10 patients were diagnosed on the basis of pathological examination of a surgical resection specimen. The median tumor size after resection was 11 cm (ranging from 2.2 to 35 cm). Two cases of recurrence, after surgical management, were observed among tumors classified as high risk of recurrence that had not benefited from adjuvant targeted therapy. Two cases of metastasis were identified. The first was colorectal GIST that metastasized to the liver and the right half-mandible and the second was a gastric GIST that metastasized to the liver.

### Therapeutic and prognostic aspects

**Fig. 3:**
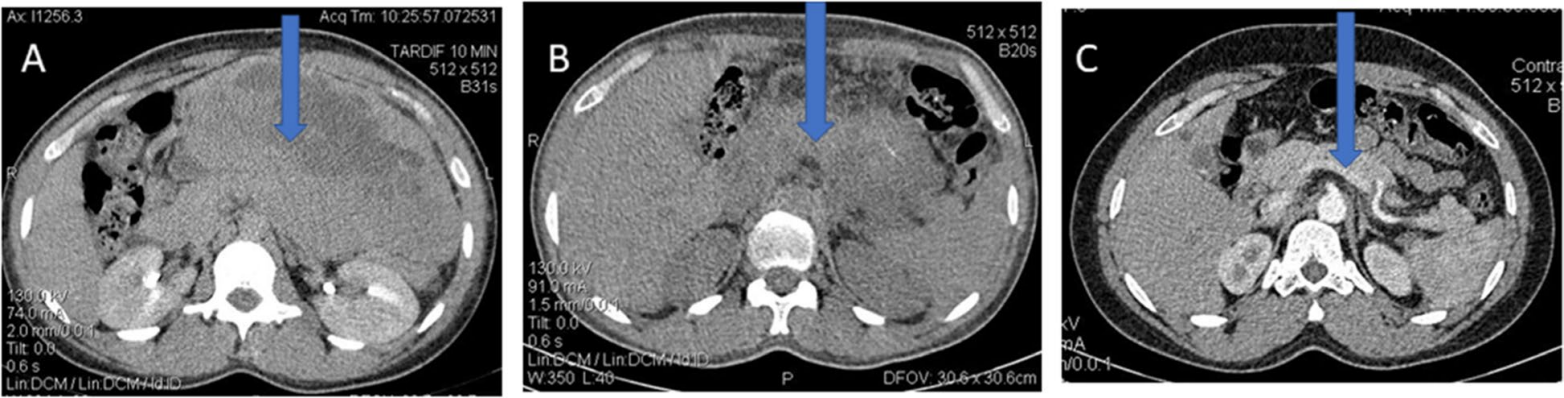
**A** Large gastric GIST (blue arrows) in a 40-year-old patient, **B** after 4 months of treatment, and **C** after 9 months of treatment

In our study, ten patients received imatinib; seven as neoadjuvant treatment and three as adjuvant treatment. The daily dose was 400 mg for all patients. The response was good for all patients with a regression of symptoms, an improvement of general condition, and a decrease in tumor size observed on CT (Fig. 3). Patients who received adjuvant imatinib (*n* = 3), did not have a recurrence. Four cases with metastatic GIST have been treated with imatinib for palliative purposes. The first was a colorectal GIST with a metastasis to the right hemi-mandible who started treatment as soon as the diagnosis was made. The second and the third were respectively a rectal and a mesentery GIST that were resected but did not receive adjuvant therapy because they were lost to follow-up. By the time they were found, their tumor had already recurred and invaded nearby organs. The fourth was a case of gastric GIST that had metastasized to the liver. Because of the delay in returning from immunohistochemistry and the presumed liver origin of the tumor, she was given sorafenib for some time before switching to imatinib once the GIST diagnosis was made.

Nine out of the fifteen patients had undergone tumor resection which was performed during open surgery. Resection margins were assessed in six cases. Tumor resection was complete (R0) in all cases. Two cases of recurrence were identified. The first was a mesentery GIST and the second was a rectal GIST. The risk of recurrence according to Joensuu's modified NIH scoring system was high in both patients. Only one of the patients died from gastrointestinal bleeding following voluntary discontinuation of his medical treatment. Patients’ information are summarized in Table 1.

**Table 1:** Summarize of results

Patients	Age (years)	Sex	Location	Tumor stage	Histology	Immuno-histo-Chemistry	Risk of recurrence	Surgery	Targeted therapy	Survival (months)	Comments
N°1	72	M	Colon and rectum	Metastatic	Fusiform	KIT+, DOG1+, CD34+	High	No	Palliative	12	Alive
N°2	40	M	Stomach	Locally advanced	Fusiform	KIT+	High	No	Neoadjuvant	9	Waiting for surgery
N°3	68	M	Stomach	Locally advanced	Fusiform	KIT+, DOG1	High	No	Neoadjuvant	6	Waiting for surgery
N°4	82	F	Mesentery	Localized	Fusiform	KIT+, DOG1+	High	Yes	Palliative (after recurrence)	36	Recurrence after 3 years
N°5	66	F	Stomach	Locally advanced	Mixed	KIT+, DOG1+	High	Yes	Adjuvant	35	Alive
N°6	52	M	Rectum	Localized	Fusiform	KIT+	High	Yes	Palliative (after recurrence)	36	Recurrence after 3 years
N°7	39	F	Jejunum	Localized	Fusiform	KIT+, DOG1+	Low	Yes	No	5	Alive
N°8	66	M	Stomach	Locally advanced	Epithelioid	KIT+, DOG1+, CD34+	High	Yes	Adjuvant	24	Alive
N°9	43	M	Omentum	Locally advanced	Fusiform	KIT+	High	Yes	Adjuvant	3	Alive
N°10	46	M	Rectum	Locally advanced	Fusiform	KIT+, DOG1+	High	No	Neoadjuvant	12	Waiting for surgery
N°11	41	M	Stomach	Locally advanced	Fusiform	KIT+	High	No	Neoadjuvant	7	Waiting for surgery
N°12	52	M	Stomach	Localized	Fusiform	KIT−, DOG1−	Very low	Yes	No	31	Alive
N°13	50	F	Stomach	Metastatic	Fusiform	KIT+, CD34+	High	No	Palliative	24	Dead
N°14	62	F	Jejunum	Locally advanced	Fusiform	KIT+, CD34+	High	Yes	Adjuvant	36	Alive
N°15	45	M	Stomach	Localized	Fusiform	KIT+, CD34+	Intermediary	Yes	No	108	Alive

## Discussion

GISTs are rare digestive tumors [1]. Fifteen cases were identified during the study period. Similar trends were seen in other African series. Only 19 cases of gastric GIST were identified in Cairo, Egypt, over a 6-year period at the National Cancer Institute; and 10 cases of GIST (all localizations included) over a 8-year period at the main hospital of Dakar, Senegal [6, 7].

The median age at diagnosis was 52 years. Similar data are reported by other series from Africa [6, 7]. The median age in the western series seems higher than ours, ranging from 65,8 to 69 years [12–14]. This could be explained by the higher age of the western population as well as their better life expectancy.

We noted in our series a male preponderance. However, GISTs usually affect both genders without predilection [3]. Other studies from Africa with a small sample size reported similar results [3, 7, 15].

Delay in care seeking after the onset of symptoms ranged from 24 h to 15 years. In case of non-emergency, patients seek health care late, probably because of their low-income status. This leads to late diagnosis which involve a higher risk of recurrence.

All patients were symptomatic, and the most frequent symptoms were abdominal pain and abdominal mass. Other African series studies have reported similar symptoms [6, 7]. Most GISTs remain silent until reaching a large size. Symptoms vary according to location and size. The main symptoms are pain and gastrointestinal bleeding [2, 3]. The delay in diagnosis in Africa has probably caused the tumors to become larger and palpable, and the patients to become symptomatic.

The stomach was the most common site for GISTs with 8 cases followed by the large bowel. Given the size of our sample, the interpretation of the frequency of locations could be biased. For instance, the second most frequent location in many studies is the small intestine [2, 3, 12, 14].

**Fig. 4:**
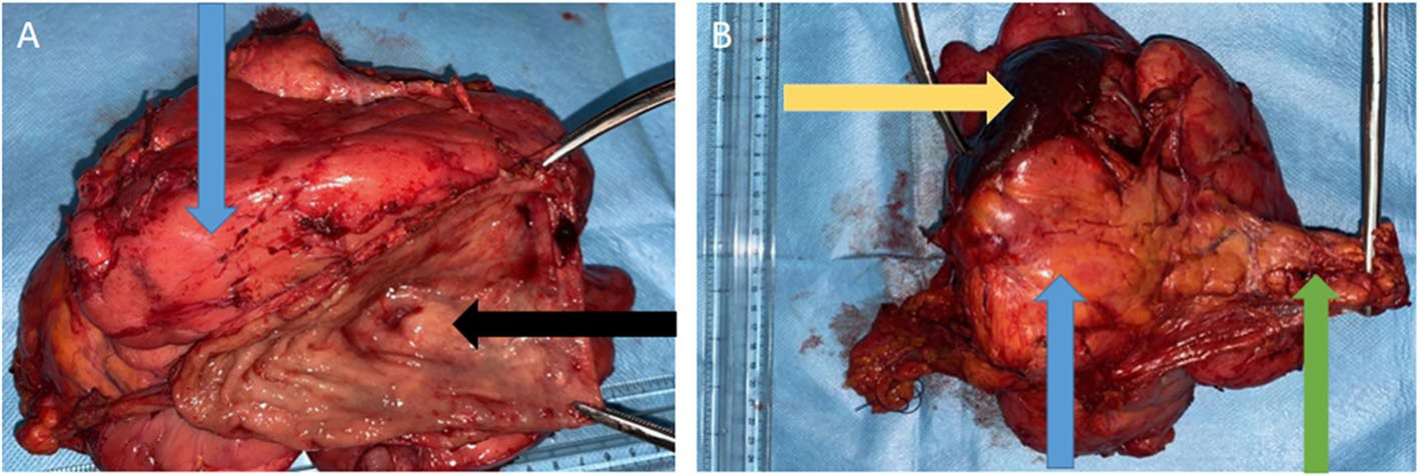
**A** Gastric mucosa with a slight nonspecific (black arrow) smooth bulge due to a large gastric GIST (blue arrow) in a 66-year-old patient. **B** Complex resection of the gastric tumor involving the spleen (yellow arrow) and the tail of the pancreas (green arrow)

In our series, endoscopy contributed to diagnosis in few cases (*n*=5). The typical endoscopic appearance of GIST is usually a nonspecific smooth bulge covered by normal mucosa [16]. The endoscopic lesions (ulcerating and ulcerative-budding lesions) in our series were probably due to tumor size. But the intraluminal lesion isn’t always correlated with tumor size. Figure 4A is an example of large gastric GIST associated with a small intraluminal lesion.

In our study, all endoscopic biopsies were conclusive. In contrast, according to many authors, the endoscopic biopsy used to be negative because it does not often reach the submucosa where the tumor is located [3, 17]. Our biopsies were conclusive probably because of endoscopic appearance of the tumors which were large with a relatively large intraluminal portion.

Laparoscopy was performed on two patients for diagnostic purposes. This allowed for adequate neoadjuvant treatment to be put in place. However, in western studies, it is most often performed for therapeutic purposes [18, 19]. In our series, the size of the tumors did not allow laparoscopic resection.

Most of the tumors resected in the study were larger than 10 cm (*n* = 6) with a median size of 11 cm. The large size of the tumors was due to the fact that patients, because of their low income, sought medical management late. Therefore, the treatment strategy was neoadjuvant imatinib therapy for at least 6 to 12 months before resection. This results in a reduction in the size of the tumor, making it easier to remove. Similar data with large tumor sizes have been reported from other low-income countries of Africa and South America [6, 20, 21]. On the contrary, studies in Iceland, Korea, and Japan have found a median tumor size of about 4 cm [14, 22]. The large tumor size is the evidence of the impact of delayed diagnosis in low-income countries like Benin. Such locally advanced tumors led to complex resection in our setting. For example, in this case, depicted in Fig. 4, a 66 years old and had gastric GIST infiltrating the abdominal wall. He received neoadjuvant imatinib with a good response. At laparotomy, a tumor invading the spleen and pancreas remained. He underwent Complex resection of the gastric tumor involving the spleen and the tail of the pancreas. Unfortunately, this is a frequent case in our practice.

We noted a strong predominance of the fusiform cell type and the expression of KIT. In fact, GISTs are often spindle-shaped types and almost always express the KIT [2, 3, 16, 23]. Almost half of the tumors (*n* = 7) expressed DOG1. DOG1 is a sensitive and specific biomarker that can contribute to the diagnosis of GIST [24]. It is important to point out that the low income of patients contributes to diagnosis delay due to the high cost of immunohistochemistry in our country. The cost of an immunohistochemistry test is 157 dollars while the guaranteed minimum wage in Benin is 70 dollars. In addition, the CNHU HKM did not have a pathology laboratory equipped for immunohistochemistry during the study period. Therefore, immunohistochemistry was performed on specimens sent abroad (mainly in France). Moreover, exams such as molecular biology are required to improve treatment. Unfortunately, these diagnostic methods are not available in most sub-Saharan African countries such as Benin.

Most of our patients were at high risk of recurrence according to Joensuu’s modified NIH scoring system. Our data are similar to those of Egypt [6]. In Iceland, Korea, and Japan, few patients presented a high risk of recurrence [14, 22]. This could be explained by the large size of the tumors in the African series due to delay in diagnosis. However, other scores such as the peripheral blood neutrophil-to-lymphocyte ratio (NLR), platelet-to-lymphocyte ratio (PLR), Onodera’s prognostic nutritional index (OPNI) and the Naples prognostic score (NPS) have recently been shown to be good predictors of outcome in GISTs preoperatively [25, 26].

The only potentially curative treatment of GISTs is a complete surgical resection [2, 3]. In situations where the tumor is locally advanced, resection is eligible if it is complete. Patients may receive adjuvant therapy (primarily imatinib in the first line and sunitinib in the second line) for up to three years, depending on their risk of recurrence. The alternative of a neoadjuvant therapy makes sense when the resection appears too complex or uncertain preoperatively. Surgery is considered when the maximum response is observed (after 6 to 12 months of treatment) [3]. Although imatinib treatment has not yet been standardized, this preoperative therapy is very effective. For example, in our study and others, it leads to a reduction in tumor size and facilitates surgical resection [27, 28].

Access to imatinib was difficult in Africa especially in Benin [7, 10]. The high cost of targeted therapies for GIST creates a significant barrier to providing cancer patients in low- and middle-income countries [11]. Until 2010 imatinib was not available in Benin [10]. A high risk of recurrence was associated to poor outcomes. But for the past five years, the GIPAP program has enabled patients with GIST to obtain imatinib free of charge. This has greatly improved the outcome of GIST in our country. However, an effort must be made to make second-line treatment, such as sunitinib and sorafenib, financially accessible. In fact, even if sorafenib is actually available in Benin, its cost (279 dollars) is prohibitive.

This study highlights the difficulty of managing GIST in low-income countries such as Benin. Its particularity lies in the diagnostic methods including histology but also immunohistochemistry. Its weaknesses are essentially the small sample size and the retrospective data collection.

## Conclusion

GISTs are rare in Cotonou. Both genders are affected with a male predominance. The median age of diagnosis was 52 years. In many low-income countries such as Benin, there is a concern of late diagnosis resulting in large tumor size. This involves a high risk of recurrence and leads to complex surgical management. The free availability of imatinib has significantly improved the outcome of GIST in low-income countries. However, an effort to reduce the cost of immunohistochemistry and ensure access to molecular biology as well as the availability of second-line treatment would ensure a better prognosis for patients.

## Data Availability

The data and materials contributing to this article may be made available upon request by sending an e-mail to the corresponding author.

## Abbreviations

CD34: Antigen CD34
CD117: Antigen CD117 ou tyrosine-protein kinase
DOG1: Discovered on GIST 1
GIPAP: Glivec International Patient Assistance Program
GIST: Gastro-intestinal stromal tumor
KIT: Tyrosin-protein kinase
NIH: National Institute of Health
